# Supplementary material for: HIVprotI: an integrated web based platform for prediction and design of HIV proteins inhibitors
Source: J Cheminform. 2018 Mar 9;10:12. doi: 10.1186/s13321-018-0266-y (PMC5845081; doi:10.1186/s13321-018-0266-y)
Supplement: Supplementary file 2 — Additional file 2. Source code of HIVProtI web server. [file 13321_2018_266_MOESM2_ESM.zip › HIVprotI_Source-code/hivprotI/weka-3-6-6/doc/weka/core/pmml/FieldMetaInfo.Optype.html]

FieldMetaInfo.Optype


---


|  |  |  |
| --- | --- | --- |
| **Overview** \| **Package** \| **Class** \| **Tree** \| **Deprecated** \| **Index** \| **Help** \| **Weka's home** | |  |
| **PREV CLASS**   **NEXT CLASS** | **FRAMES**    **NO FRAMES**     **All Classes** |  |
| SUMMARY: NESTED \| ENUM CONSTANTS \| FIELD \| METHOD | DETAIL: ENUM CONSTANTS \| FIELD \| METHOD |  |


---


## weka.core.pmml Enum FieldMetaInfo.Optype

```
java.lang.Object
  java.lang.Enum<FieldMetaInfo.Optype>
      weka.core.pmml.FieldMetaInfo.Optype
```

**All Implemented Interfaces:**: java.io.Serializable, java.lang.Comparable<FieldMetaInfo.Optype>

**Enclosing class:**: FieldMetaInfo

---

``` public static enum FieldMetaInfo.Optype extends java.lang.Enum<FieldMetaInfo.Optype> ```

Enumerated type for the Optype

---

**Enum Constant Summary**

`CATEGORICAL`

`CONTINUOUS`

`NONE`

`ORDINAL`


| **Method Summary** | |
| --- | --- |
| `java.lang.String` | `toString()` |
| `static FieldMetaInfo.Optype` | `valueOf(java.lang.String name)`             Returns the enum constant of this type with the specified name. |
| `static FieldMetaInfo.Optype[]` | `values()`             Returns an array containing the constants of this enum type, in the order they are declared. |

**Methods inherited from class java.lang.Enum**

`compareTo, equals, getDeclaringClass, hashCode, name, ordinal, valueOf`

**Methods inherited from class java.lang.Object**

`getClass, notify, notifyAll, wait, wait, wait`

**Enum Constant Detail**

### NONE

```
public static final FieldMetaInfo.Optype NONE
```

---


### CONTINUOUS

```
public static final FieldMetaInfo.Optype CONTINUOUS
```

---


### CATEGORICAL

```
public static final FieldMetaInfo.Optype CATEGORICAL
```

---


### ORDINAL

```
public static final FieldMetaInfo.Optype ORDINAL
```


**Method Detail**

### values

```
public static FieldMetaInfo.Optype[] values()
```

:   Returns an array containing the constants of this enum type, in
    the order they are declared. This method may be used to iterate
    over the constants as follows:

    ```
    for (FieldMetaInfo.Optype c : FieldMetaInfo.Optype.values())
        System.out.println(c);
    ```

    :   **Returns:**: an array containing the constants of this enum type, in the order they are declared

---


### valueOf

```
public static FieldMetaInfo.Optype valueOf(java.lang.String name)
```

:   Returns the enum constant of this type with the specified name.
    The string must match *exactly* an identifier used to declare an
    enum constant in this type. (Extraneous whitespace characters are
    not permitted.)

    :   **Parameters:**: `name` - the name of the enum constant to be returned. **Returns:**: the enum constant with the specified name **Throws:**: `java.lang.IllegalArgumentException` - if this enum type has no constant with the specified name: `java.lang.NullPointerException` - if the argument is null

---


### toString

```
public java.lang.String toString()
```

:   **Overrides:**: `toString` in class `java.lang.Enum<FieldMetaInfo.Optype>`


---


|  |  |  |
| --- | --- | --- |
| **Overview** \| **Package** \| **Class** \| **Tree** \| **Deprecated** \| **Index** \| **Help** \| **Weka's home** | |  |
| **PREV CLASS**   **NEXT CLASS** | **FRAMES**    **NO FRAMES**     **All Classes** |  |
| SUMMARY: NESTED \| ENUM CONSTANTS \| FIELD \| METHOD | DETAIL: ENUM CONSTANTS \| FIELD \| METHOD |  |


---
